# Schistosome Eggs Impair Protective Th1/Th17 Immune Responses Against *Salmonella* Infection

**DOI:** 10.3389/fimmu.2018.02614

**Published:** 2018-11-14

**Authors:** Gabriele Schramm, Abdulhadi Suwandi, Alibek Galeev, Samriti Sharma, Janin Braun, Anne-Kathrin Claes, Peter Braubach, Guntram A. Grassl

**Affiliations:** ^1^Experimental Pneumology, Research Center Borstel, Airway Research Center North, Member of the German Center for Lung Research (DZL), Borstel, Germany; ^2^Institute of Medical Microbiology and Hospital Epidemiology and German Center for Infection Research (DZIF), Partner Site Hannover, Hannover Medical School, Hannover, Germany; ^3^Institute for Experimental Medicine, Christian-Albrechts-University of Kiel, Kiel, Germany; ^4^Research Center Borstel, Borstel, Germany; ^5^Institute for Pathology, Hannover Medical School, Hannover, Germany

**Keywords:** co-infection, *Schistosoma mansoni*, *Salmonella* Typhimurium, Th17, Th1, immunoregulation, IPSE/alpha-1

## Abstract

Countries with a high incidence of helminth infections are characterized by high morbidity and mortality to infections with intracellular pathogens such as *Salmonella*. Some patients with *Salmonella*-*Schistosoma* co-infections develop a so-called “chronic septicemic salmonellosis,” with prolonged fever and enlargement of the liver and spleen. These effects are most likely due to the overall immunoregulatory activities of schistosomes such as induction of Tregs, Bregs, alternatively activated macrophages, and degradation of antibodies. However, detailed underlying mechanisms are not very well investigated. Here, we show that intraperitoneal application of live *Schistosoma mansoni* eggs prior to infection with *Salmonella* Typhimurium in mice leads to an impairment of IFN-γ and IL-17 responses together with a higher bacterial load compared to *Salmonella* infection alone. *S. mansoni* eggs were found in granulomas in the visceral peritoneum attached to the colon. Immunohistological staining revealed IPSE/alpha-1, a glycoprotein secreted from live schistosome eggs, and recruited basophils around the eggs. Noteworthy, IPSE/alpha-1 is known to trigger IL-4 and IL-13 release from basophils which in turn is known to suppress Th1/Th17 responses. Therefore, our data support a mechanism of how schistosomes impair a protective immune response against *Salmonella* infection and increase our understanding of helminth-bacterial co-infections.

## Introduction

In developing countries with a high incidence of helminth infections, co-infections with other microorganisms such as human immunodeficiency virus (HIV), *Mycobacterium tuberculosis*, and *Salmonella enterica* are frequent (1). Compared to infection caused by a single pathogenic species, co-infection can alter the immune response of the host and thus affect the outcome of each infection. Co-infection of *Schistosoma mansoni* and *S. enterica* are very common in endemic areas in Africa and South-East Asia. Some patients co-infected with schistosomes and *Salmonella* develop a so-called “chronic septicemic salmonellosis,” with prolonged fever and enlargement of the liver and spleen (2, 3). Co-infected animals have greater bacteremia and more persistent local systemic infection in the liver and spleen compared to *Salmonella*-only infected animals (1).

Non-typhoidal *Salmonella* (NTS) serovars, such as *S. enterica* serovar Typhimurium (*S*. Typhimurium) usually cause self-limiting diarrhea in immunocompetent hosts (4). Infection leads to Toll-like receptor (TLR) activation, production of inflammatory cytokines such as IL-6, IL-1β, TNF-α, CCL2, IFN-γ, and IL-17, and neutrophil and macrophage recruitment. However, in small children, immunocompromised hosts (e.g., HIV-infected individuals), and in helminth-infected persons, bacteremia develops with higher bacterial loads in the mesenteric lymph nodes (mLN) and spleen.

Schistosomes have strong immunomodulatory effects on the immune system of their hosts (5). Characteristic for schistosome infection is the induction of a strong Th2 response with high IL-4 and IL-13 production, IgE synthesis, and eosinophilia during schistosomiasis (6). Th2 cytokines induce the alternative activation of macrophages and suppress pro-inflammatory cytokine release such as IL-1ß, IL-6, and IL-17, thus directing the immune response toward wound healing and tissue repair (7). The result is the induction of a modified or regulated Th2 response, during the chronic phase of infection (8). Epidemiological studies and animal experiments have shown that schistosome infections can protect against excessive inflammation caused by asthma, allergies, and autoimmune diseases (9). On the other hand, their immunoregulatory effect can impair immune responses necessary to combat other pathogens and to develop a protective antibody response (9). Schistosome eggs are mainly responsible for the immunomodulatory effects (10). Each couple of *Schistosoma mansoni* produces ~300 eggs per day which are deposited in the mesenteric veins. Half of the eggs are carried away with the blood stream and embolize in the liver. The other half migrate through the intestinal tissue, preferentially through Peyer's patches lymphoid tissue, toward the gut lumen (11). During this process the eggs induce granulomatous inflammation (6, 7) and release immunomodulatory products, e.g., the egg antigens IPSE/alpha-1 and omega-1 (12–14), which are in close contact with immune cells in the granuloma (15) as well as the Peyer's patches.

To investigate the influence of schistosome eggs on an infection with *Salmonella* we used a well-established mouse model for intestinal *Salmonella* infection (16, 17). *S*. Typhimurium is a facultative intracellular pathogen that causes enterocolitis in humans, while in mice it causes a typhoid-like disease with little intestinal inflammation. However, pretreatment of mice with streptomycin leads to a higher expansion of *Salmonella* in the intestine and severe intestinal inflammation resembling the pathology of human enterocolitis (16, 18). Since the eggs of schistosomes have immunomodulatory abilities, we aimed in this study at investigating the effect of schistosome eggs on concurrent *Salmonella* infection. Therefore, live *S. mansoni* eggs purified from infected hamster livers were intraperitoneally injected into mice one week before *Salmonella* infection. One day, two weeks, and five weeks following *Salmonella* infection, the immune response of the mice was investigated with respect to histopathological changes, intestinal, and systemic bacterial load, immune cell recruitment, and cytokine production. We demonstrate that *S. mansoni* eggs lead to the suppression of protective cytokine responses against *Salmonella* and subsequent persistence of the bacteria.

## Materials and methods

### Animals and infection

Eight week old C57Bl/6J mice were purchased from Charles River (Sulzbach, Germany) and housed in the animal facility at the Research Center Borstel, Germany. Mice were injected with 5,000 eggs intraperitoneally. Seven days later, mice were given 20 mg streptomycin by oral gavage for efficient intestinal colonization with *Salmonella*. *S*. Typhimurium SL1344 Δ*aroA* (19) were grown overnight in Luria-Bertani broth at 37°C with shaking. Twenty-four hours after streptomycin treatment, mice were infected with 3 x 10^6^ *S*. Typhimurium SL1344 Δ*aroA* suspended in 100 μL HEPES buffer (100 mmol/l, pH 8.0). Control mice received 100 μL HEPES buffer alone.

### Ethics statement

All experiments were conducted consistent with the ethical requirements and approval of the Animal Care Committee of the Ministry of Energy, Agriculture, the Environment and Rural Areas of Schleswig-Holstein, Germany and in direct accordance with the German Animal Protection Law. The protocols were approved by the Ministry of Energy, Agriculture, the Environment and Rural Areas of Schleswig-Holstein, Germany [Protocol#: V244-7224.121.3 (38-4/11)].

### Isolation of *S. mansoni* eggs from infected hamster livers

Livers of *S. mansoni*-infected hamsters (kindly provided by Prof. Dr. C. G. Grevelding, Institute for Parasitology, Justus-Liebig-University, Giessen) were homogenized by short pulses with an Ultra-Turrax (Model TP18/10, Janke & Kunkel GmbH, IKA-Labortechnik, Staufen) in ice-cold HEPES buffer (137 mM NaCl, 12 mM HEPES, 5.5 mM glucose, 3.8 mM Na_3_PO_4_, 2.7 mM KCl, pH 7.5). Eggs were released from the tissue by digestion with 0.05% collagenase (Invitrogen), 1.5 mg/ml hyaluronidase (Roth), and 1.5 mg/ml dispase (Invitrogen) in HEPES buffer (10 ml/g liver) for 3 h at 37°C. Cell/egg suspensions were cooled on ice and centrifuged at 1,500 rpm (Heraeus Varifuge 3.0R) for 2 min at 4°C. Eggs were washed several times with ice-cold HEPES buffer, changing the centrifugation speed from 1,500 rpm to 1,200 rpm and finally to 1,000 rpm until eggs were free of any remaining cells and debris. Purity was controlled under a microscope. The final washing step was performed with phosphate-buffered saline (PBS), pH 7.5. Purified eggs were counted and eggs were kept in PBS at 4°C in the dark until application into mice.

### Histopathological scoring

Assessment of severity of pathological changes in the intestine was performed as described (20). Briefly, tissue was fixed in 10% formalin prior to embedding in paraffin. Five micro meters sections of the cecum and colon were stained with hematoxylin and eosin (H&E). Pathological scores were determined separately for the lumen, surface epithelium, mucosa, and submucosa as follows. Lumen: necrotic epithelial cells (scant, 1; moderate, 2; dense, 3) and polymorphonuclear leukocytes (PMNs) (scant, 1; moderate, 2; dense, 3). Surface epithelium: desquamation (patchy, 1; diffuse 2), ulceration (absent, 0; present, 1). Mucosa: crypt abscesses (rare, <15%, 1; moderate, 15 to 50%, 2; abundant, >50%, 3); mononuclear cell infiltrate (1 small aggregate, 0; >1 aggregate, 1; large aggregates plus increased single cells, 2); inflammatory cell infiltrate (scant, 1; moderate, 2; dense, 3). Submucosa: mononuclear cell infiltrate (1 small aggregate, 0; >1 aggregate, 1; large aggregates plus increased single cells, 2); inflammatory cell infiltrate (scant, 1; moderate, 2; dense, 3); edema, (mild, 0; moderate 1; severe 2). Total severity score is the sum of the four sub-scores.

### Quantitative real-time polymerase chain reaction (qPCR)

RNA was extracted from mouse colon tissue using the High Pure RNA Tissue Kit (Roche). RNA was reverse transcribed into cDNA using the cDNA Synthesis Kit (Roche) according to the manufacturer's instructions. Quantitative real-time PCR (pPCR) was performed with SYBR-Green Mastermix (Roche) and the following gene-specific primers (Table 1) on a BioRad C1000/CFX96 real time machine. Data were normalized to house-keeping genes *Gapdh* and *Hprt1* and fold change was calculated using the ΔΔC_t_ method.

**Table 1:** Primers used in this study.

**Primer name**	**Sequence**
*Hprt1 f*	AGTGTTGGATACAGGCCAGAC
*Hprt1 r*	CGTGATTCAAATCCCTGAAGT
*Gapdh f*	ATTGTCAGCAATGCATCCTG
*Gapdh r*	ATGGACTGTGGTCATGAGCC
*Ifng f*	TCAAGTGGCATAGATGTGGAAGAA
*Ifng r*	TGGCTCTGCAGGATTTTCATG
*Il13 f*	AGACCAGACTCCCCTGTGCA
*Il13 r*	TGGGTCCTGTAGATGGCATTG
*Ccl2 f*	CCTGCTGTTCACAGTTGCC
*Ccl2 r*	ATTGGGATCATCTTGCTGGT
*Il17a f*	GCTCCAGAAGGCCCTCAGA
*Il17a r*	AGCTTTCCCTCCGCATTGA
*Tnfa f*	CCACCACGCTCTTCTGTCTAC
*Tnfa r*	AGGGTCTGGGCCATAGAACT

### Immunohistology and histochemistry

Tissues were fixed in formalin for 24 h before they were embedded in paraffin. Five micro meters sections were deparaffinized and rehydrated. Antigen retrieval was done in a rice steamer using sodium citrate buffer (10 mM, pH 6.0) for 30 min. Immunohistochemical staining of the sections was performed with a monoclonal antibody against IPSE/alpha-1 [clone 74 1G2 (12), dilution 1:100]. Binding was visualized by use of the M.O.M. ImmPress Polymer HRP Kit (Biozol) and Vector NovaRed as substrate (Vector Laboratories) according to the manufacturer‘s instruction. Mayer‘s hematoxylin (Merck) was used for counter staining. Pictures were taken with an Olympus BX51 microscope. For immunofluorescence staining, sections were stained against following antigens: mMCP-8 (Tug8, Biolegend, 1:250), myeloperoxidase (MPO, Thermo Scientific, 1:200), *Salmonella* (BD Biosciences, 1:100), and IL-13 (Santa Cruz, 1:50) at 4°C overnight followed by secondary fluorescently labeled antibodies (Invitrogen, 1:1,000) for 1 h at room temperature. As negative controls for staining, primary antibodies were omitted to exclude unspecific staining. For the detection of neutrophils, paraffin embedded tissue sections (5 μm thick) were deparaffinized and rehydrated. Staining was done using Naphthol-AS-D-Chloracetate esterase kit (Sigma-Aldrich) according to manufacturer's instructions. Representative images are shown and were obtained using an Axioimager (Zeiss) microscope.

### Statistics

Statistical analysis was performed using GraphPad Prism 7 software package (GraphPad Software, San Diego, CA). Student's *t*-test was used when two groups were compared. One-way analysis of variance (ANOVA) with Tukey's multiple comparison post-test was used to determine significance between three or more data sets. Data are presented as means +SD or as box and whiskers plots as indicated in the figure legends. A *p*-value smaller than 0.05 was considered statistically significant. Colony forming units (cfu) were logarithmically transformed and subsequently analyzed by one-way analysis of variance (ANOVA) with Tukey's multiple comparison post-test.

## Results

### Treatment of mice with schistosome eggs leads to a higher colonization with *S*. Typhimurium

**Figure 1:**
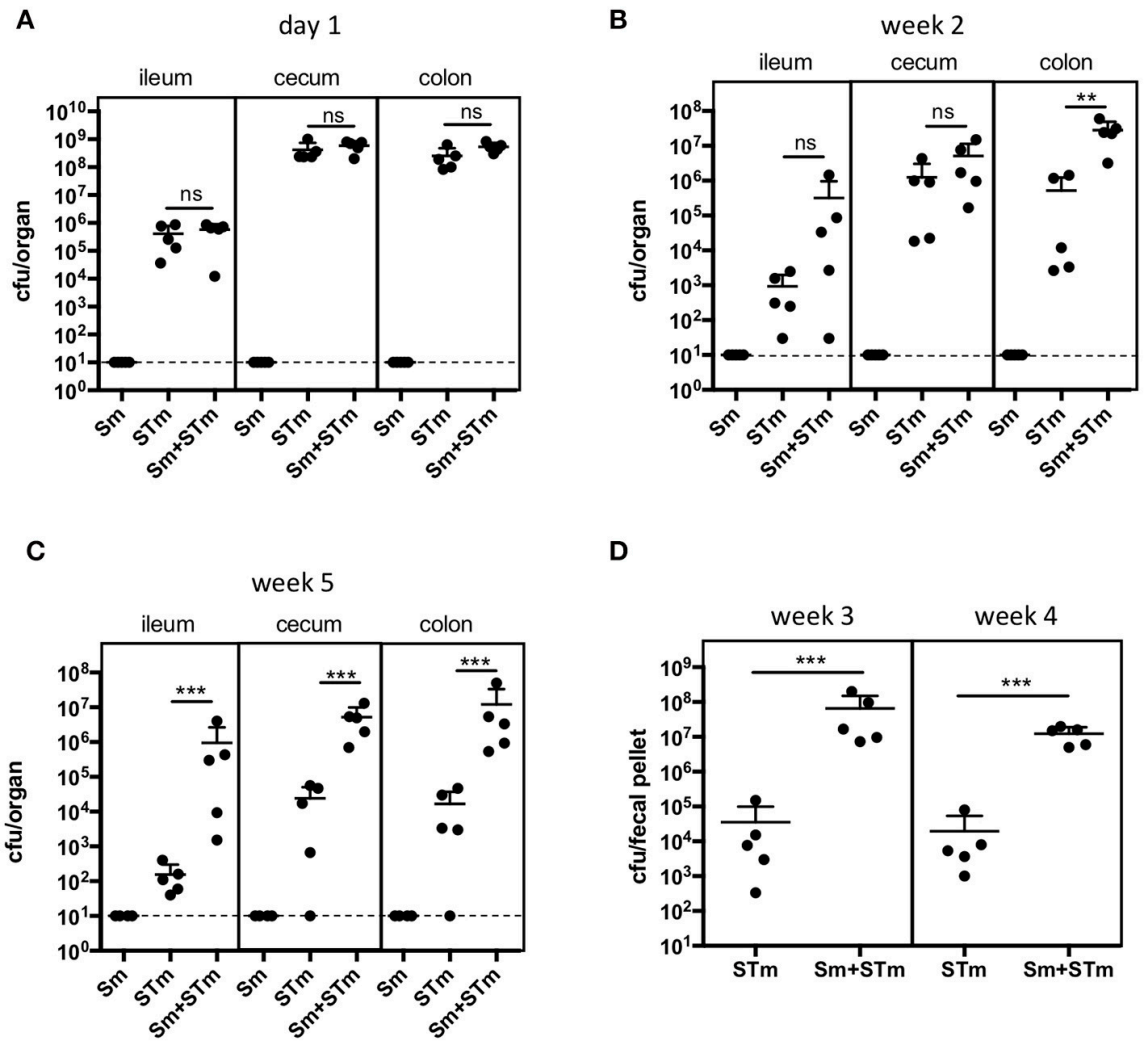
*S. mansoni* eggs (Sm) impair clearance of *Salmonella* (STm) infection. Mice were injected intraperitoneally with Sm and 8 days later they were orally infected with STm. Mice were sacrificed on day 1 **(A)**, week 2 **(B)**, and week 5 **(C)** p.i.. Feces were collected three and four weeks p.i.. **(D)**. Each data point represents one animal and the means + SD are shown (*n* = 5–10 mice per group). Dashed line indicates detection limit. Data were analyzed using one-way ANOVA with Tukey's post-test **(A-C)** or Student's *t*-test **(D)**. ***p < 0.001; **p < 0.01; ns, *p* ≥ 0.05 (not significant).

In order to assess the effect of schistosome eggs (Sm) on the outcome of infection with *S*. Typhimurium (STm), we injected eggs of *S. mansoni* purified from infected hamster liver intraperitoneally into C57Bl/6J mice and, 8 days later, mice were infected with STm. Bacterial burden in intestinal and systemic tissues was analyzed one day, two weeks, and five weeks post infection (p.i.) with STm. Sm had no effect on early STm colonization on day one p.i. (Figure 1A). However, 2 weeks p.i. significantly more STm were recovered from the colon of mice which had previously received Sm (Sm+STm) compared to mice that were only infected with STm (Figure 1B). This effect became stronger at later time points as seen in bacterial recovery in intestinal tissues 5 weeks p.i. (Figure 1C) and tracked in feces at weeks three and four p.i. (Figure 1D). However, we did not see any significant differences in colonization of the mLN, liver, or spleen (data not shown). These data suggest that schistosome eggs either enhance *Salmonella* colonization of the intestine or impair clearance of *Salmonella*.

### Schistosome eggs increase *salmonella*-induced inflammation in the luminal compartment but decrease mucosal inflammation

**Figure 2:**
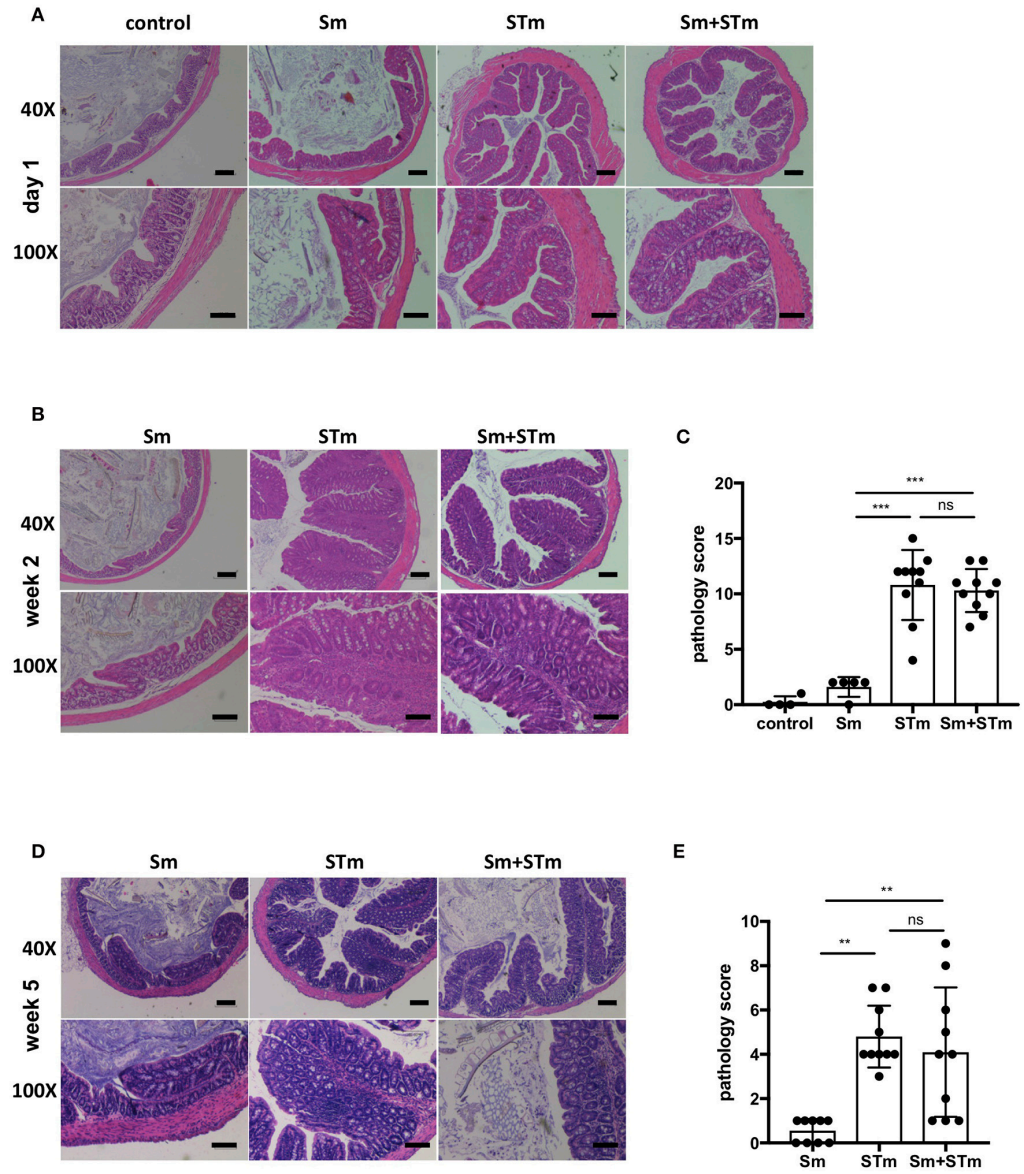
*S. mansoni* eggs (Sm) modulate *Salmonella*-induced intestinal pathology. Histopathological changes in the lumen, surface epithelium, mucosa, and submucosa were analyzed in H&E-stained sections of the proximal colon. **(A)** H&E-stained colon sections on day 1 p.i. with STm. **(B)** H&E-stained colon sections 2 weeks p.i.. **(C)** Pathology scores of week 2 p.i.. **(D)** H&E-stained colon sections 5 weeks p.i.. **(E)** Pathology scores of week 5 p.i.. Each data point represents one animal and means + SD are shown (*n* = 5–10 mice per group). Scale bars 200 μm for 40X and 100 μm of 100X magnifications. Data were analyzed using one-way ANOVA with Tukey's post-test. ***p < 0.001; **p < 0.01; ns, *p* ≥ 0.05 (not significant).

**Figure 3:**
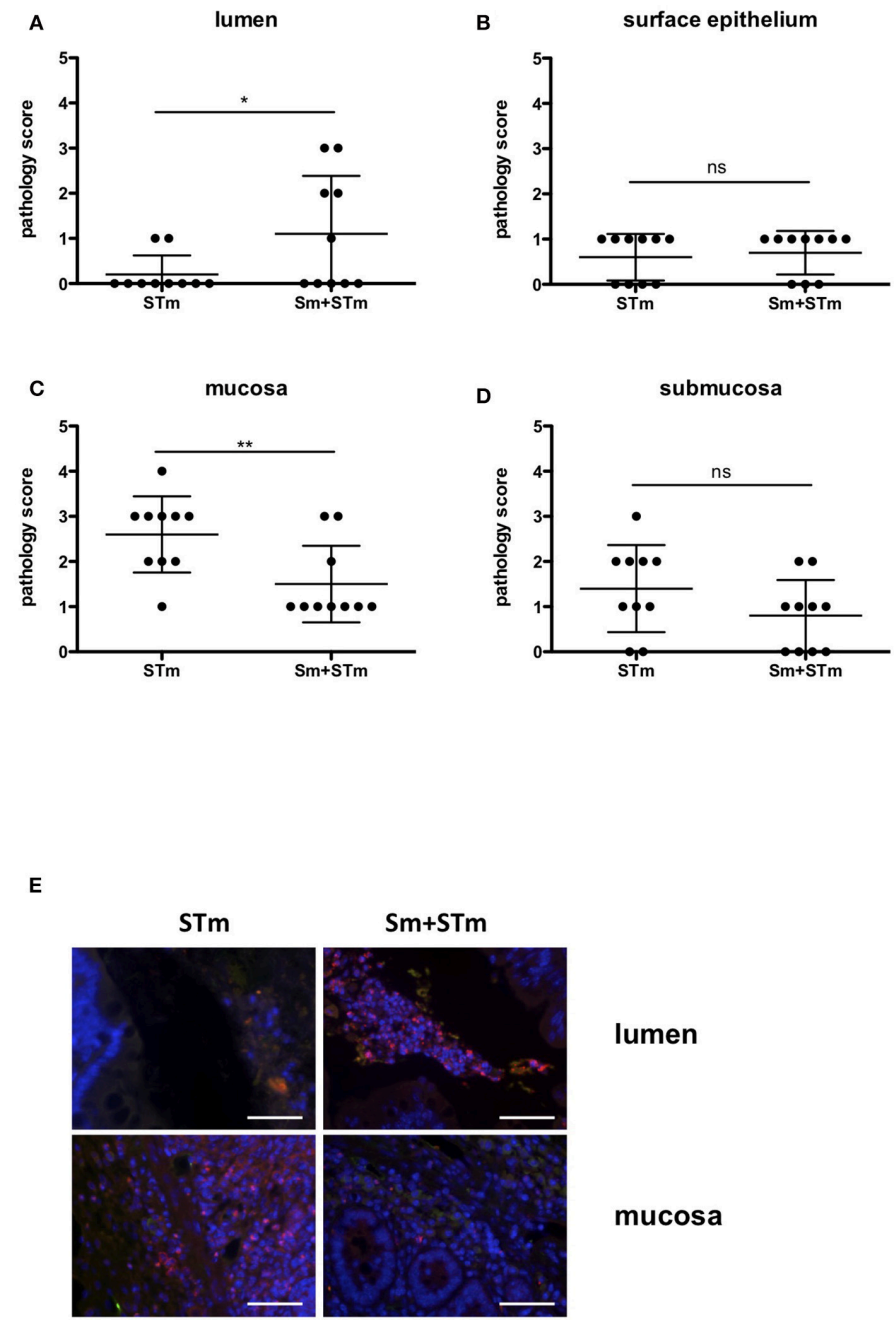
Accumulation of neutrophils upon treatment with *S. mansoni* eggs (Sm) and infection with *S*. Typhimurium (STm). **(A–D)** Pathology scores of the different areas at week 5 p.i.. **(E)** Intestinal tissue sections were stained with antibodies against MPO to visualize neutrophils. Note the accumulation of MPO positive cells in the lumen of Sm+STm-infected mice compared to only a few MPO positive cells in STm-infected mice (upper panels). In contrast, MPO positive cells were found in the mucosa of STm-infected mice but only very few in Sm+STm-infected mice (lower panels). No MPO positive cells were visible in sections from control mice or mice injected with Sm alone. Red, MPO; blue, DAPI. Scale bar 50 μm. Data were analyzed using Student's *t*-test. **p < 0.01; *p < 0.05; ns,*p* ≥ 0.05 (not significant).

Next, we assessed the intestinal pathology caused by Sm, STm infection, or Sm + STm co-infection. Pathological changes were given a score for their severity in the lumen, surface epithelium, mucosa, and submucosa as described in Material and Methods. While mice treated with only Sm did not show any apparent intestinal histopathological changes, we found strong inflammatory changes in the cecum and colon of STm-infected mice. On day 1 p.i. only mild pathology was detected in the colon of STm- and Sm+STm-infected mice (Figure 2A). However, two and five weeks p.i. there were strong inflammatory changes visible in the colons of STm- and Sm+STm-infected mice (Figures 2B–E). Despite much higher bacterial burdens in Sm+STm-infected mice at week 5 p.i., no significant differences were observed in overall pathology compared to STm-infected mice (Figure 2D). However, when the pathological changes in the various parts of the colon wall were analyzed, more necrotic epithelial cells and more inflammatory cells in the gut lumen of Sm+STm-infected mice compared to STm-infected mice were seen (Figure 3A). In contrast, inflammation of the mucosa was significantly less severe in Sm+STm-infected mice (Figure 3C). No significant differences in the surface epithelium (Figure 3B) or in the submucosa (Figure 3D) were apparent between the groups, although there was a trend toward attenuated inflammation in the submucosa of Sm+STm-infected mice compared to STm-infected mice. As the group of mice with the higher inflammatory score in the lumen had a lower inflammatory score in the mucosa, the overall pathology score was similar between both STm- and Sm+STm-infected mice (Figure 2E).

*Salmonella*-induced inflammation is characterized by a strong influx of neutrophils. Staining for myeloperoxidase (MPO), a marker for neutrophils, revealed that at week two p.i. both STm- and Sm+STm-infected mice showed strong inflammation with massive accumulation of neutrophils in the lumen and the mucosa. Consistently, at this time point there was no difference in pathology caused by STm or Sm+STm infection. However, five weeks p.i. STm-infected mice still showed a strong accumulation of neutrophils in mucosa and submucosa, with only few neutrophils in the intestinal lumen (Figure 3E, left panels). In contrast, Sm+STm-infected mice at this same late time showed only few neutrophils in mucosa and submucosa, but strong accumulation in the lumen (Figure 3E, right panels). These results indicate a down-regulation of inflammation in mucosa and submucosa of Sm+STm-infected mice by the action of the schistosome eggs.

### Intraperitoneally-injected schistosome eggs induce granuloma formation

**Figure 4:**
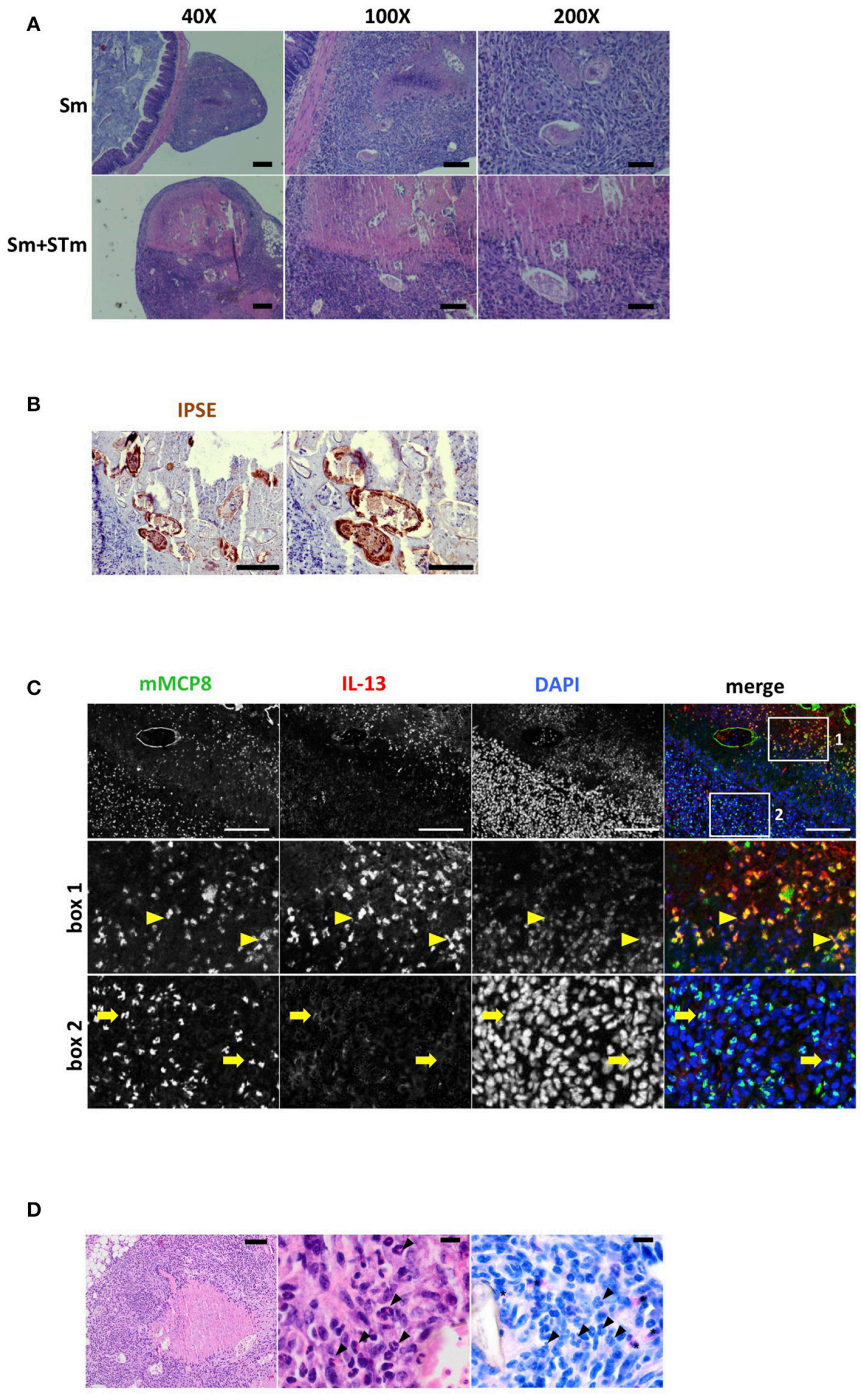
*S. mansoni* eggs (Sm) induce granuloma formation in the visceral peritoneum. **(A)** H&E-stained sections of Sm-induced granulomas in the visceral peritoneum attached to the colon. Scale bars 200 μm for 40X, 100 μm for 100X, and 50 μm for 200X magnifications. **(B)** Sections of intestinal tissue were stained with antibodies against IPSE/alpha-1 (brown). Scale bars 100 μm (left panel) and 50 μm (right panel). **(C)** IL-13 producing basophils were found in the granuloma; basophils (mMCP8, green); IL-13 (red); nuclei (DAPI, blue). Scale bar 100 μm. Note the autofluorescence of the Sm shell (green). Second row: magnification of the area indicated by box 1 containing the majority of IL-13 producing basophils. Bottom row: magnification of the area indicated by box 2 containing mainly basophils which do not produce IL-13. **(D)** H&E stains with necrotizing granuloma containing numerous *S. mansoni* eggs with central necrosis in the visceral peritoneum (left panel). The granuloma contains numerous eosinophilic granulocytes (middle panel, arrowheads). Chloroacetate esterase (CAE) staining identifies scattered neutrophilic granulocytes in the granuloma (right panel, asterisks) while eosinophilic granulocytes remain negative (right panel, arrowheads). Scale bars are 100 μm in left panel and 10 μm in middle and right panel, respectively.

During our experiments, we observed granulomatous tissue formation in the visceral peritoneum attached to the proximal part of the colon which contained the schistosome eggs (Figure 4A). Schistosome eggs produce and secrete IPSE/alpha-1. We evaluated IPSE/alpha-1 production by immunohistochemical staining with a monoclonal anti-IPSE/alpha-1 antibody. As shown in Figure 4B we found strong staining for IPSE/alpha-1 in the subshell area of the eggs and in the surrounding area in the granuloma. We did not detect any IPSE/alpha-1 staining in the adjacent intestinal tissue which was also devoid of schistosome eggs. Furthermore, we found basophils, eosinophils and a few scattered neutrophils within the granuloma (Figures 4C,D). Basophils were not detected in the neighboring part of the intestine. Since IPSE/alpha-1 triggers the release of IL-4 and IL-13 from basophils, we stained for these cytokines. Unfortunately, we were not able to establish a specific immunostaining protocol for IL-4. However, we found IL-13 produced by basophils within the granuloma which were located near to the eggs (Figure 4C, box 1, arrows). However, we did not observe IL-13 positive staining in basophils located in the outer parts of the granuloma, away from the eggs (Figure 4C, box 2, arrowheads).

### IL-17 response to *S*. Typhimurium infection is dampened by schistosome eggs

**Figure 5:**
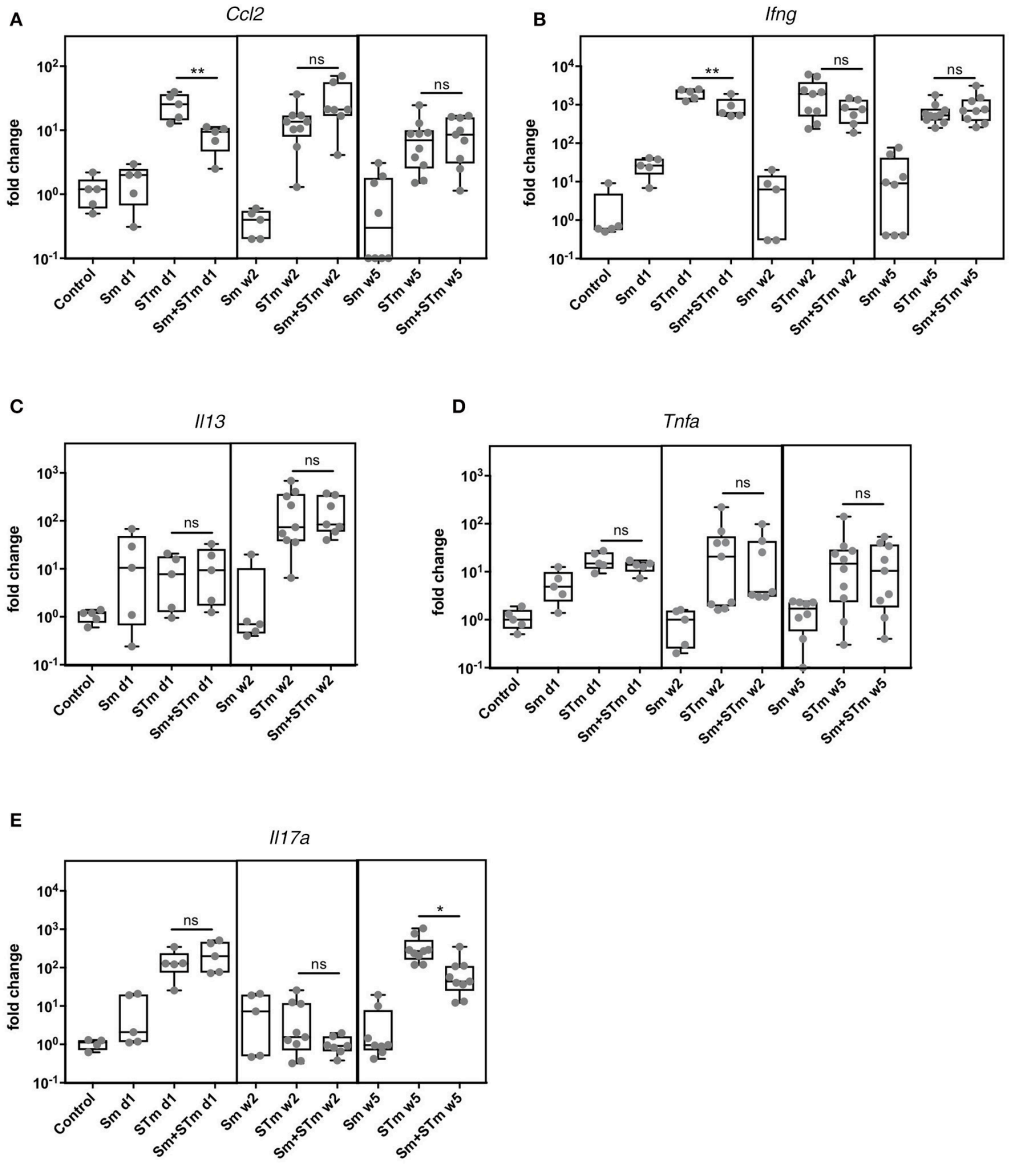
*S. mansoni* eggs (Sm) impair the STm-induced IL-17 response. RNA was isolated from intestinal tissue at day 1, week 2, and week 5 p.i.. STm infection upregulated cytokine responses including **(A)** Ccl2, **(B)** Ifng, **(C)** Il13, **(D)** Tnfa and **(E)** Il17a at all time points. Sm significantly downregulated expression of *Ifng* and *Ccl2* at day 1 p.i. and *Il17a* at week 5 p.i. while the other cytokines tested were not affected. Each data point represents one animal and box and whiskers indicating the minimum and maximum values are shown (*n* = 5–10 mice per group). Data were analyzed using one-way ANOVA with Tukey's post-test. Data analyses are only depicted for comparison of STm with Sm+STm groups. **p < 0.01; *p < 0.05; ns, *p* ≥ 0.05 (not significant).

To address whether schistosome eggs can modulate the inflammatory response during STm infection we analyzed gene expression in the intestinal tissue one day, two weeks, and five weeks p.i. by qPCR. Colon tissue adjacent to the histological sections was taken for RNA isolation. Of note, this tissue did not contain any granulomas. Sm alone had no effect on the expression of any of the cytokines in the intestine. Infection of mice with STm caused an upregulation of *Ccl2, Tnfa, Il17a*, and *Ifng* at all-time points investigated (Figure 5). On day one p.i. mice that were pre-treated with schistosome eggs (Sm+STm) had a diminished *Ccl2* and *Ifng* response, while the other cytokines were not affected (Figure 5). *Il13* was only induced at day one and week two p.i. (Figure 5C) and was not detectable at week five p.i. (not shown). Five weeks p.i., *Il17a* was strongly upregulated in STm-infected mice and *Il17a* expression was significantly reduced in Sm+STm-infected mice (Figure 5E) suggesting that schistosome eggs dampen the STm-induced IL-17 response.

## Discussion

Co-infection with two different pathogens commonly occurs in endemic countries. However, our understanding how one infection alters the outcome of another infection is limited. Most infection models focus on one pathogen at a time. To study the mutual influence of two pathogens on the immune response, we chose *Salmonella* Typhimurium and *Schistosoma mansoni*, since these pathogens are commonly causing co-infections. Infections with non-typhoidal *Salmonella* strains are usually limited to acute infections in immunocompetent persons and are associated with high levels of neutrophils and the cytokines IFN-γ and IL-17 (21). However, sometimes they can cause persistent infections (22). This is often linked to antibiotic treatment or co-infections with other pathogens (23, 24). In contrast to *Salmonella*, the parasitic worm *S. mansoni* causes persistent chronic infections. While the adult worms in the mesenteric veins elicit only limited immune reactions, *S. mansoni* eggs induce a strong Th2 response with high titers of IL-4, IL-5, and IL-13 and formation of granulomas rich in Th2 cells, basophils, eosinophils, alternatively activated macrophages, and Tregs (10, 25). This “modified” Th2 response seems to be protective for the host. In contrast, a Th1/Th17 immune response, found to be protective in *Salmonella* infection, results in exaggerated inflammation and early death of certain schistosome-infected individuals or schistosome-infected mice with a Th17-biased immune response (26).

Here, we applied *S. mansoni* eggs instead of a complete adult worm infection. We aimed to investigate the contribution of the schistosome eggs to the modulation of the immune response in a concomitant *Salmonella* infection, as the schistosome eggs are the major immunomodulatory life cycle stage of schistosomes (10). We demonstrate that mice injected with schistosome eggs one week before infection with *S*. Typhimurium were higher colonized with *S*. Typhimurium compared to mice infected with *Salmonella* alone. The higher loads of *Salmonella* in the schistosome eggs-treated mice correlated with an increase in inflammatory cells in the intestinal lumen. In contrast, inflammation in the mucosa was weaker in these mice compared to mice only infected with *Salmonella*. It was recently shown that application of *Schistosoma* egg antigens can ameliorate inflammation in a T cell transfer model of colitis by enhancing Th2 and diminishing Th17 responses (27). Here, we found that schistosome eggs impaired Th1 responses at early time points post infection with *Salmonella* and Th17 responses at later time points post infection. *Salmonella* infection causes a strong Th1/Th17 response and while these responses are required for successful elimination of the pathogen (21), they also contribute to tissue damage and inflammation (28). Co-infection with *Schistosoma* has been long known to contribute to impaired clearance of *Salmonella* in human patients (3, 29). One mechanism for *Salmonella* persistence during co-infection could be the intimate attachment of *Salmonella* pili to glycosylated structures on the tegument of adult schistosome worms (30). However, this has so far only been demonstrated *in vitro* and not in *in vivo* co-infections. Here, we present evidence for an immune-mediated mechanism for bacterial persistence, as there are no adult worms present in our *Salmonella* infection model with schistosome egg injection. Our observations are in agreement with findings that blocking of the Th17 response results in reduced neutrophil recruitment and diminished bacterial clearance (31). In SIV-infected rhesus macaques with mucosal IL-17 deficiency a high systemic dissemination of *S*. Typhimurium was observed (32). Likewise, mice co-infected with the intestinal helminth *Heligmosomoides polygyrus* and *S*. Typhimurium develop severe intestinal inflammation, with reduced neutrophil recruitment, reduced inflammatory cytokine production (IL-17, IL-22, IL-23), and higher bacterial load in feces, cecum, mLN, and spleen compared to mice infected with *Salmonella* alone (33). Together, these results demonstrate that a functional Th17 response is crucial to keep *Salmonella* colonization under control and finally to clear the infection. *H. polygyrus* has also been shown to alter the intestinal metabolome thereby affecting *Salmonella* virulence and leading to increased colitis (34). In line with the reduced Th1/Th17 response, we observed reduced neutrophil infiltration and diminished tissue destruction in the mucosa and submucosa in mice treated with schistosome eggs. As a consequence however, these mice could not control *Salmonella* growth and persistence in the intestinal lumen. Although neutrophils are important for clearing bacterial infections, *Salmonella* has evolved mechanisms to evade killing by neutrophils (35). It has also been shown that *Salmonella* can reside and survive in the intestinal lumen attached to and within neutrophils (36). Furthermore, *Salmonella* can take advantage of the inflammatory milieu in the intestinal lumen to grow and outcompete other bacteria (37, 38).

The Th2 cytokines IL-4 and IL-13 are known to dampen the production of pro-inflammatory cytokines such as IL-1β, IL-6, TNF-α, and IL-17 (39–41). The major egg antigen secreted from live schistosome eggs, IPSE/alpha-1, triggers basophils to release IL-4, and IL-13 (12, 42). Recently, we demonstrated the presence of IPSE/alpha-1 and basophils in schistosome egg granulomas and showed that IPSE/alpha-1-triggered basophil IL-4 and IL-13 inhibited pro-inflammatory cytokine release from LPS-activated human monocytes *in vitro* (43). In the present study we detected IPSE/alpha-1, recruited basophils, and IL-13 in egg-induced granulomas attached to the colon of co-infected mice by immunohistological staining. IL-13 co-localized with basophils near the schistosome eggs and not with basophils in the outer regions of the granuloma. This indicates that Th2 cytokine release from basophils was induced by schistosome eggs i.e., their secretions. Since IPSE/alpha-1 represents more than 80% of the egg secretions (44) and is the only factor secreted from schistosome eggs that is able to induce IL-13 release from basophils (12) it is most likely that this factor is responsible for Th2 cytokine release and subsequent reduction of Th1/Th17 responses. In addition, we observed early dampening of CCL2 production which could result in impaired chemoattraction of inflammatory monocytes and thus adds to an inadequate control of *Salmonella* colonization.

Schistosome eggs must carefully navigate the host's inflammatory response. On one hand they must cause some tissue damage and inflammation to enable egress of the eggs to complete the schistosome life cycle, on the other hand they protect the host tissue from egg-released products (proteases, RNases), and promote tissue repair and wound healing to prevent excessive inflammation (45). To this end, they induce a typical Th2 granuloma, containing Th2 cells, basophils, eosinophils, and alternatively activated macrophages, but few neutrophils. Immunocompromised mice or mice with a Th17-biased immune response develop smaller granulomas containing higher numbers of neutrophils and develop lesions within surrounding liver cells (45, 46). Seki et al. showed that IL-4 and IL-13 inhibited neutrophil influx into egg granulomas in the liver of mice infected with *S. japonicum* (47). In line, we detected elevated numbers of neutrophils in the gut tissue of Sm+STm-infected mice outside the egg granulomas, but eosinophils and only few scattered neutrophils within the egg granulomas.

Not surprisingly, *Salmonella* affects the outcome of a concomitant schistosome infection as well. In a recent study it was shown that *S*. Typhimurium elicits a protective effect on mice infected with *S. japonicum* (48). In this study, mice with a fully established (five weeks) *S. japonicum* infection were co-infected with wild-type *S*. Typhimurium for nine days. *Salmonella* co-infection on top of an infection with *S. japonicum* was beneficial, as it reduced *S. japonicum* worm burden, and subsequently liver egg burden and enhanced the survival rate of the co-infected mice. Our study differs from the above mentioned as we did not use a patent schistosome infection, but we applied one dose of 5,000 eggs of *S. mansoni* intraperitoneally. This is a relatively moderate treatment compared to a *S. japonicum* infection with deposition of 3,000 eggs per day per worm couple. Furthermore, we used a *Salmonella* infection with an attenuated strain, which allowed us to follow immune responses for up to 35 days post infection. Thus, our experimental set-up was not suitable for investigating the effect of a *Salmonella* infection on the outcome of a schistosome infection with regard to worm burden and survival rate, but allowed to investigate the modulation of the immune response to *Salmonella* infection by schistosome eggs. In the study by Zhu and colleagues co-infection of *S. japonicum* with *S*. Typhimurium led to an exacerbated IFN-γ response but to an attenuated IL-4 response (48). In contrast, in our study using intraperitoneal injection of *S. mansoni* eggs, we find a decrease in *S*. Typhimurium infection-induced IFN-γ presumably due to the action of the products released by schistosome eggs. This may reflect the differences between infection with the entire worm and injection of purified schistosome eggs or/and the usage of different mouse strains. While we used C57Bl/6 mice, the above-mentioned study was performed with BALB/c mice, known to have a Th2-biased immune response. While a moderate Th2 response protects the host from excessive inflammation, a strong Th2 response might lead to high worm burden and egg release, and heavy scar formation and fibrosis. Thus, schistosome-infected Th2-prone individuals might profit from a Th17-inducing co-infection, and the outcome of a co-infection does not only depend on the infecting pathogens but also on the pre-existing immune situation of the host.

In summary, we demonstrate that schistosome eggs modulate the immune response to *Salmonella* infection affecting *Salmonella*-induced pathology and leading to *Salmonella* persistence, presumably by dampening Th1/Th17 responses of the host.

## Data availability statement

All datasets generated for this study are included in the manuscript.

## Author contributions

GS and GG planned experiments, performed experiments, analyzed data, wrote manuscript. AS, AG, SS, JB, AKC, and PB performed experiments, analyzed data, edited, and approved manuscript.

### Conflict of interest statement

The authors declare that the research was conducted in the absence of any commercial or financial relationships that could be construed as a potential conflict of interest.
